# From Trust to Betrayal: Imposter Nurses and Nursing Quackery in Nigeria, Their Threat to Patient Safety, and the Role of Professionalism: A Cross‐Sectional Survey

**DOI:** 10.1002/nop2.70707

**Published:** 2026-07-17

**Authors:** Benjamin Olusola Ajibade, Isaiah Dada Owoeye, Abiola Ogunniyi

**Affiliations:** ^1^ Faculty of Health and Wellbeing, School of Healthcare and Nursing Sciences Northumbria University Newcastle UK; ^2^ Nursing Image and Standards Support Initiative of Nigeria (NISSIN) Nurses House Abeokuta Nigeria; ^3^ School of Nursing University of the Western Cape Cape Town South Africa; ^4^ Neuropsychiatric Hospital, Aro Abeokuta Nigeria

**Keywords:** ethical practice, healthcare regulation, Nigeria, nursing quackery, patient safety, professionalism, trust

## Abstract

**Background:**

Nursing quackery; the unlicensed practice of nursing by unqualified individuals poses a major threat to patient safety and nursing integrity in Nigeria. Despite a regulatory council, systemic enforcement failures continue to enable imposters to operate countrywide.

**Aim:**

To examine perceptions of nursing quackery in Nigeria, assess registered nurses' professional values, professionalism, trust, and ethical practice, and identify predictors of quackery awareness.

**Methods:**

A cross‐sectional survey of 618 registered nurses recruited across all six Nigerian geopolitical zones used multistage sampling. Data were collected using the Nigerian Nursing Professionalism and Quackery Assessment Tool (NNPQAT), adapted from the Nurses' Professional Values Scale‐Revised (NPVS‐R), Hall's Professionalism Scale, the Trust in Nurses Scale, and the Ethical Behaviour Rating Scale. Chi‐square tests, Spearman's rho correlations, and multiple linear regression were performed using SPSS version 29.

**Results:**

Participants demonstrated high professional values (*M* = 4.76, SD = 0.44), professionalism (*M* = 4.76, SD = 0.58), ethical practice (*M* = 4.84, SD = 0.59), and trust (*M* = 4.53, SD = 0.59). Quackery awareness was widespread (*M* = 4.53, SD = 0.58); 96.1% agreed quackery threatens patient safety and 97.1% agreed it damages public trust. Awareness did not vary by region (*χ* ^2^ = 2.25, *p* = 0.814) or facility type (*χ* ^2^ = 0.18, *p* = 0.915). Ethical practice (*ρ* = 0.421, *p* < 0.001) and trust (*ρ* = 0.361, *p* < 0.001) were the strongest correlates. Regression identified ethical practice (*β* = 0.541, *p* < 0.001) and trust (*β* = 0.215, *p* < 0.001) as the only significant predictors (*R* ^2^ = 0.511); years of experience, professionalism, region, and facility type were non‐significant.

**Conclusions:**

Nigerian registered nurses exhibit strong professional values, yet quackery persists as a system‐level failure rooted in regulatory weakness rather than individual professional deficit. Ethical practice functions as professional vigilance, heightening sensitivity to quackery. Strengthening Nursing and Midwifery Council of Nigeria (NMCN) enforcement, investing in ethics education, and developing public awareness campaigns are essential to protect patients and preserve professional integrity.

**Implications for Nursing Practice or Education:**

These findings call for heightened regulatory vigilance among registered nurses, nurse educators, and healthcare administrators in identifying and reporting suspected nursing quackery. Pre‐registration and continuing professional development curricula should embed ethics education that develops professional vigilance, recognising that ethical orientation, not seniority or years of experience, is the strongest driver of quackery awareness. Clinical and educational leaders should establish routine credential verification practices at ward and facility level, supported by safe, protected reporting mechanisms for nurses who identify unqualified colleagues. Nurse educators are encouraged to incorporate public awareness content into community health teaching, equipping the public to verify nursing credentials and recognise warning signs of fraudulent practice. These actions are relevant across all facility types and regions, given the uniform national distribution of quackery awareness identified in this study.

**No Patient or Public Contribution:**

This study did not involve patients, service users, caregivers, or members of the public in its design, conduct, data analysis, or manuscript preparation. The study population comprised registered nursing professionals only, recruited as expert informants on the phenomenon of nursing quackery in Nigeria. Patient and public involvement was not applicable to this study.

## 1 Introduction

Nursing professionalism is foundational to safe, effective, and ethical healthcare delivery. Grounded in values such as trust, accountability, competence, and adherence to ethical standards, professionalism sustains public confidence in health systems (Cao et al. 2023). Yet in many low‐ and middle‐income countries (LMICs), including Nigeria, these foundations face a persistent and serious threat: the proliferation of healthcare quackery. Broadly defined as the deliberate practice of unqualified individuals providing clinical care for financial or personal gain, quackery represents a globally recognised public health crisis (Jarvis 1999; Amir‐Azodi et al. 2024).

Within nursing, quackery takes a particularly alarming form. Nursing quackery refers to individuals who falsely present themselves as trained and licensed nurses while lacking the requisite education, certification, and professional approval to practise. These imposters frequently operate in underserved communities, private clinics, or informal healthcare settings where regulatory oversight is weakest and qualified professional access most limited. Consequences include misdiagnosis, inappropriate treatment, delayed referrals, and, in severe cases, preventable morbidity and mortality (Imoleayo 2023).

The persistence of quackery globally has been attributed to an interplay of socio‐economic, cultural, and systemic factors. A recent scoping review highlighted that poverty, healthcare workforce shortages, weak regulatory systems, and low public health literacy together create environments in which unqualified practitioners can thrive (Amir‐Azodi et al. 2024). In many settings, patients prioritise affordability and geographic proximity over professional credentials, inadvertently sustaining the demand for quack services. These dynamics are especially pronounced in sub‐Saharan Africa, where health system inequities and unequal distribution of skilled workers compound vulnerabilities.

Nigeria presents a particularly acute case. Despite the existence of the NMCN, mandated to regulate nursing education and practice, enforcement challenges persist. Inadequate monitoring capacity, institutional gaps in licensing verification, and limited regulatory reach have contributed to the proliferation of unlicensed practitioners (Aborode et al. 2021). Socio‐cultural perceptions of healthcare, including entrenched trust in informal providers and community‐based care traditions, further complicate elimination efforts.

The implications extend well beyond individual patient harm. Trust is a fundamental component of the nurse–patient relationship, influencing patient satisfaction, treatment adherence, and health outcomes (Radwin and Cabral 2010). When unqualified individuals masquerade as registered nurses and deliver substandard care, the resulting adverse experiences erode public trust in the profession as a whole, creating a damaging paradox: qualified registered nurses, despite their adherence to high professional standards, suffer reputational harm from the actions of imposters.

Furthermore, nursing quackery challenges the professional and ethical identity of registered nurses. Professionalism encompasses not merely technical competence but a commitment to ethical practice, self‐regulation, and accountability (Hall 1968; Weis and Schank 2009). Quackery blurs the boundary between legitimate and illegitimate practice, undermining these principles and placing additional burdens on qualified registered nurses, who must navigate the consequences of diminished trust and rectify harm caused by imposters.

Despite its significance, empirical research specifically examining nursing quackery in Nigeria, particularly from the perspective of practising registered nurses, remains scarce. This study addresses that gap. It was conceived and initiated by the Nursing Image and Standards Support Initiative of Nigeria (NISSIN), a professional advocacy organisation dedicated to promoting nursing standards, protecting the image of the profession, and supporting evidence‐based regulatory reform across Nigeria. This study represents NISSIN's first formal research project, undertaken without external funding, reflecting the initiative's commitment to generating the evidence base needed to drive systemic change.

### 1.1 Theoretical Framework

This study is underpinned by Professionalism Theory (Hall 1968) and Trust Theory in Healthcare (Radwin and Cabral 2010), applied in combination to interpret findings across multiple dimensions.

Hall's (1968) Professionalism Theory conceptualises professionalism as a multidimensional construct comprising autonomy, commitment to public service, self‐regulation, and a sense of vocation. In nursing, this framework illuminates how professional values and behaviours distinguish qualified practitioners from imposters, and how self‐regulation functions as a safeguard against fraudulent practice.

Complementing this, Trust Theory in healthcare (Radwin and Cabral 2010) emphasises that trust is built on perceived competence, honesty, and ethical conduct. When quackery undermines these perceptions, it disrupts the relational foundation of care. Integrating these frameworks allows this study to examine nursing quackery not only as a regulatory and clinical issue but also as a threat to professional identity, public trust, and the relational infrastructure of healthcare.

### 1.2 Aims and Objectives

The primary aim of this study is to examine the phenomenon of nursing quackery in Nigeria and its implications for professionalism, patient safety, and public trust. Specific objectives, all addressed within this cross‐sectional quantitative phase, are to:

- Assess the levels of professional values, professionalism, trust, and ethical practice among registered nurses in Nigeria.
- Examine the prevalence of quackery awareness and reported exposure to harm among registered nursing professionals.
- Investigate whether quackery awareness and encounters with harm differ by geopolitical region and facility type.
- Explore the relationships between professional and ethical constructs and quackery awareness.
- Identify the independent predictors of quackery awareness scores among Nigerian registered nurses.

## 2 Methods

### 2.1 Study Design

A quantitative cross‐sectional survey design was employed to examine the prevalence, perceptions, and professional correlates of nursing quackery among registered nurses in Nigeria. This design was selected because it allows data collection from a large, geographically distributed population at a single point in time, enabling simultaneous assessment of multiple variables, group comparisons, and the identification of predictors—directly addressing all five study objectives. A cross‐sectional approach is appropriate for Objectives 1 and 2 (description of professional constructs and quackery prevalence), Objectives 3 (group‐level comparisons by region and facility type using chi‐square analysis), Objective 4 (bivariate relationships using correlational analysis), and Objective 5 (independent predictors using multiple linear regression) (Creswell 2014; Field 2013). This paper reports findings from the quantitative component of a broader mixed‐methods study.

### 2.2 Study Setting

The study was conducted across Nigeria's six geopolitical zones: North Central, North East, North West, South East, South South, and South West. These regions differ substantially in healthcare infrastructure, workforce distribution, regulatory capacity, and access to services, making geographic spread across all zones essential. Registered nurses were drawn from urban, semi‐urban, and rural healthcare settings to capture this diversity.

### 2.3 Participants

The target population comprised registered nurses and nursing professionals actively practising in Nigeria. Eligible participants included staff nurses, nurse managers, nurse educators, and advanced nursing practitioners. Inclusion criteria required: registration with the NMCN or equivalent professional recognition; current active practice in a Nigerian healthcare setting; and willingness to provide informed consent. Student nurses, retired nurses not actively practising, and non‐nursing healthcare workers were excluded.

### 2.4 Sample Size and Sampling

A total of 618 registered nurses provided complete valid responses from 627 recruited, yielding a response rate of 98.6%. A multistage sampling strategy was employed to ensure geographic coverage across all six geopolitical zones. In Stage 1, Nigeria was stratified into its six geopolitical zones. In Stage 2, healthcare facilities were purposively selected within each zone to include diverse facility types: public government hospitals, private healthcare institutions, and non‐governmental organisation (NGO)‐run facilities. In Stage 3, individual participants were recruited using purposive and convenience sampling within selected facilities, targeting registered nurses currently practising across all grade levels and specialties.

It is important to note that while multistage sampling and geographic stratification enhance the representativeness of the sample, Stage 3 convenience sampling within facilities means that findings cannot be generalised to all registered nurses in Nigeria with the confidence afforded by probability sampling methods. The sample provides broad coverage across all six geopolitical zones but should be understood as a cross‐sectional sample rather than a statistically representative sample of the national registered nursing workforce. The sample size supported the statistical power required for regression modelling with multiple predictors (Kline 2015).

### 2.5 Instrument

Data were collected using the NNPQAT, a structured questionnaire developed through adaptation of four validated international instruments:

- NPVS‐R (Weis and Schank 2009; *α* ≈ 0.92).
- Hall's Professionalism Scale (Hall 1968; *α* = 0.75–0.89).
- Trust in Nurses Scale (TIN; Radwin and Cabral 2010; *α* > 0.80).
- Ethical Behaviour Rating Scale (EBRS; Humphries et al. 2019; *α* ≈ 0.85).

Context‐specific items were added to capture quackery awareness and impact within the Nigerian healthcare context. The instrument comprised seven sections: (A) demographic characteristics, (B) professional values (7 items; PV1–PV7), (C) professionalism (5 items; PRO8–PRO12), (D) trust in nursing (5 items; TRUST13–TRUST17), (E) ethical practice (4 items; ETHIC18–ETHIC21), (F) quackery awareness and impact (8 items; QUACK22–QUACK29), and (G) open‐ended qualitative questions (not analysed in this paper). All scale items used a 5‐point Likert format (1 = Strongly Disagree to 5 = Strongly Agree). Content validity was assessed through expert review, and a pilot study confirmed acceptable reliability (Cronbach's *α* ≥ 0.70 across all subscales).

### 2.6 Data Collection

Data were collected between August 2025 and January 2026 using a structured online questionnaire administered via Google Forms (Google LLC 2025). Google Forms was selected for its ease of distribution, automated data capture, accessibility across mobile and desktop devices (Eysenbach 2004), cost‐effectiveness, and capacity for broad geographic reach across multiple regions simultaneously (Evans and Mathur 2018). The survey link was distributed electronically through professional nursing networks, ward and unit managers, social media platforms, institutional contacts, and NISSIN contacts within participating facilities across all six geopolitical zones. The use of online survey methods among Nigerian nursing professionals has been established as both feasible and methodologically appropriate for this educated professional population, given increasing smartphone penetration and internet access (Adeloye et al. 2022). Participants received an information sheet explaining the study purpose, voluntary participation, right to withdraw, and confidentiality assurances. Completion of the questionnaire constituted informed consent. No identifying information was collected; responses were anonymised and stored in password‐protected systems accessible only to the research team. To minimise duplicate responses, participants were clearly instructed to complete the survey only once; this instruction appeared both in the participant information sheet and on the survey opening page.

### 2.7 Data Analysis

Data were analysed using SPSS version 29. Descriptive statistics (frequencies, percentages, means, and standard deviations) were calculated for all demographic and scale variables. Subscale scores were computed as mean scores across constituent items (higher scores indicate stronger professional values and awareness of quackery). Three levels of inferential analysis were conducted:

- Chi‐square tests of independence (*χ* ^2^) to examine associations between geopolitical region and quackery awareness, and between facility type and encounters with patient harm attributable to quackery. Cramér's *V* was calculated as an effect size measure.
- Spearman's rank correlation coefficients (rho) were used to explore bivariate relationships between subscale scores and quackery awareness, given the ordinal and non‐normally distributed nature of the scale data.
- Multiple linear regression with the overall quackery awareness score as the dependent variable. Predictors included years of nursing experience, facility type, geopolitical region (dummy‐coded, South West as reference), professionalism score, ethical practice score, and trust score. Prior to regression, assumptions were verified: normality of residuals was assessed using P–P plots; homoscedasticity was examined using scatterplots of standardised residuals against predicted values; and multicollinearity was assessed using Variance Inflation Factor (VIF) values, all of which fell below 3.0, indicating no problematic multicollinearity (Myers 1990; Tabachnick and Fidell 2019). Although residuals showed some non‐normality (skewness = −1.25, kurtosis = 7.83) consistent with a ceiling effect in the outcome variable, bootstrapped confidence intervals (1000 samples) were computed as a robustness check and yielded findings consistent with ordinary least squares (OLS) estimates. Adjusted *R* ^2^, standardised coefficients (*β*), and bootstrapped 95% confidence intervals are reported. Statistical significance was set at *p* < 0.05.

The study adhered to STROBE (Strengthening the Reporting of Observational Studies in Epidemiology) guidelines for cross‐sectional research, and to the CHERRIES (Checklist for Reporting Results of Internet E‐Surveys) guidelines for online survey methodology (Eysenbach 2004). See File S1—STROBE and CHERRIES checklist.

### 2.8 Ethical Considerations

Ethical approval was obtained from the Babcock University Health Research Ethics Committee, Nigeria (Approval Number: BUHREC/2025/1127/24; approved 31st July 2025). As this study was conducted entirely in Nigeria by the Nursing Image and Standards Support Initiative of Nigeria (NISSIN), of which the corresponding author (Benjamin Ajibade) is Research Lead, with no UK‐based data collection, processing, or direct researcher contact with participants taking place within UK jurisdiction, separate ethical approval from a UK institution was not required and was not sought. All participants provided informed consent. Confidentiality and anonymity were maintained throughout. Participants were informed of their right to withdraw at any stage without penalty. Data were stored securely in password‐protected systems accessible only to the research team.

## 3 Results

### 3.1 Participant Characteristics

A total of 618 registered nurses provided complete data. The mean age was 43.44 years (SD = 10.8; range: 20–73 years), reflecting a predominantly mid‐career and experienced sample. The majority were female (*n* = 481, 77.8%; male: *n* = 137, 22.2%). Table 1 provides a full overview of demographic characteristics.

**TABLE 1:** Demographic characteristics of participants (*N* = 618).

Variable	Category	*n* (%)
Age	Mean 43.44 (SD 10.8)	Range: 20–73 years
Gender	Female	481 (77.8%)
	Male	137 (22.2%)
Highest qualification	BSc nursing	393 (63.1%)
	MSc nursing	81 (13.0%)
	RN only	63 (10.1%)
	RM licence	28 (4.5%)
	RN + postbasic certificate	43 (6.9%)
	PhD	11 (1.8%)
Years of practice	Mean 17.78 (SD 10.7)	Range: 1–48 years
Current position	Nurse manager/matron	247 (40.0%)
	Staff nurse	210 (34.0%)
	Nurse educator	62 (10.0%)
	Other/senior roles	76 (16.0%)
Facility type	Public (government)	542 (86.4%)
	Private	56 (8.9%)
	NGO	16 (2.6%)
Geopolitical zone	South West	306 (48.8%)
	South East	96 (15.3%)
	North Central	89 (14.2%)
	North West	48 (7.7%)
	North East	47 (7.5%)
	South South	34 (5.4%)
Location	Urban	424 (67.6%)
	Semi‐Urban	106 (16.9%)
	Rural	88 (14.0%)

In terms of highest qualification, the majority held a Bachelor of Nursing Science (BSc Nursing; *n* = 393, 63.1%), with MSc Nursing (*n* = 81, 13.0%) and Postgraduate Diploma qualifications collectively representing 14.8% of the sample, indicating a well‐educated professional group. A smaller proportion held the basic Registered Nurse (RN) qualification alone (*n* = 63, 10.1%) or a Registered Midwife licence (*n* = 28, 4.5%).

Participants reported a mean of 17.78 years of nursing practice (SD = 10.7; range: 1–48 years), confirming substantial collective experience. By position, nurse managers and matrons constituted the largest group (*n* = 247, 40.0%), followed by staff nurses (*n* = 210, 34.0%), nurse educators (*n* = 62, 10.0%), and other senior or specialist roles (*n* = 76, 16.0%).

The majority were employed in public sector facilities (*n* = 542, 86.4%), with private (*n* = 56, 8.9%) and NGO (*n* = 16, 2.6%) settings making up the remainder. Geopolitically, the South West zone was overrepresented (*n* = 306, 48.8%), which is acknowledged as a study limitation; the remaining five zones each contributed between 5.4% and 15.3% of the sample. Most registered nurses practised in urban settings (*n* = 424, 67.6%), with semi‐urban (*n* = 106, 16.9%) and rural (*n* = 88, 14.0%) representation also present.

### 3.2 Professional Values

Registered nurses demonstrated consistently high professional values, with an overall mean score of 4.76 (SD = 0.44). All items exceeded 90% agreement. Table 2 presents item‐level results. The highest‐rated items reflected fundamental nursing commitments: belief in maintaining patient trust (PV3: *M* = 4.86, SD = 0.46; 97.6%) and justice and fairness in healthcare delivery (PV6: *M* = 4.86, SD = 0.41; 97.4%). Patient advocacy, while still high, was the lowest‐rated item (PV4: *M* = 4.43, SD = 0.73; 90.0%).

**TABLE 2:** (a) Professional values scale results (*N* = 618). (b) Professionalism scale results (*N* = 618). (c) Trust in Nursing and Ethical Practice Scale results (*N* = 618).

Item	*M* (SD)	Agreed (%)
*(a)*		
PV3: Maintaining patient trust is essential	4.86 (0.46)	97.6%
PV6: Justice and fairness in healthcare delivery	4.86 (0.41)	97.4%
PV2: Care without discrimination	4.84 (0.54)	96.5%
PV5: Continuing education to improve practice	4.82 (0.44)	97.5%
PV1: Uphold patient confidentiality	4.80 (0.47)	98.6%
PV7: Maintaining high standards under workplace pressure	4.70 (0.57)	96.7%
PV4: Advocate for patients' needs (lowest item)	4.43 (0.73)	90.0%
Overall scale	4.76 (0.44)	—
*(b)*		
PRO10: Nursing as a calling/vocation	4.74 (0.55)	95.0%
PRO9: Member of professional nursing organisation	4.72 (0.58)	95.7%
PRO8: Commitment to self‐regulation	4.65 (0.62)	94.0%
PRO12: Public service orientation	4.58 (0.66)	92.1%
PRO11: Supporting colleague accountability (lowest item)	4.36 (0.81)	88.9%
Overall Scale	4.76 (0.58)	—
*(c)*		
**Trust in nursing**	**Scale *M* (SD): 4.53 (0.59)**	
TRUST13: Patients have confidence in my clinical skills	4.69 (0.52)	97.7%
TRUST17: Team trust improves patient outcomes	4.68 (0.53)	96.6%
TRUST14: Patients trust my clinical judgement	4.58 (0.62)	94.8%
TRUST16: I trust my colleagues to maintain high standards	4.46 (0.71)	90.1%
TRUST15: Colleagues act in patients' best interests (lowest)	4.19 (0.78)	84.5%
**Ethical practice**	**Scale *M* (SD): 4.84 (0.59)**	
ETHIC21: Ethical practice is essential to maintain public trust	4.92 (0.42)	98.3%
ETHIC20: Refuse to participate in unethical practice	4.88 (0.48)	97.3%
ETHIC19: Willing to report unqualified colleagues	4.82 (0.56)	91.7%
ETHIC18: Adhere to NMCN professional standards	4.73 (0.64)	93.0%

### 3.3 Professionalism, Trust in Nursing, and Ethical Practice

Table 2b presents item‐level results for the Professionalism subscale. Professionalism scores were high overall (*M* = 4.76, SD = 0.58). The highest scores were for viewing nursing as a calling (PRO10: *M* = 4.74, SD = 0.55; 95.0%) and professional organisation membership (PRO9: *M* = 4.72, SD = 0.58; 95.7%). Supporting colleague accountability was the lowest‐rated item (PRO11: *M* = 4.36, SD = 0.81; 88.9%).

Table 2c presents Trust in Nursing and Ethical Practice results. Trust scores were high (*M* = 4.53, SD = 0.59). Registered nurses strongly endorsed patient confidence in clinical skills (TRUST13: *M* = 4.69, SD = 0.52; 97.7%) and team trust improving outcomes (TRUST17: *M* = 4.68, SD = 0.53; 96.6%). Trust in colleagues to act in patients' best interests was the lowest‐rated item (TRUST15: *M* = 4.19, SD = 0.78; 84.5%). Ethical practice demonstrated the highest overall mean (*M* = 4.84, SD = 0.59), with near‐universal agreement for belief that ethical practice maintains public trust (ETHIC21: *M* = 4.92, SD = 0.42; 98.3%), refusal to participate in unethical practice (ETHIC20: *M* = 4.88, SD = 0.48; 97.3%), and willingness to report unqualified colleagues (ETHIC19: *M* = 4.82, SD = 0.56; 91.7%).

### 3.4 Quackery Awareness and Impact

Quackery‐related items revealed both high awareness and widespread exposure (overall *M* = 4.53, SD = 0.58). Table 3 presents item‐level results. The highest‐rated items concerned normative beliefs: quackery damages public trust in nursing (QUACK24: *M* = 4.82, SD = 0.66; 97.1%) and poses a serious threat to patient safety (QUACK23: *M* = 4.77, SD = 0.76; 96.1%). Items measuring direct personal exposure showed greater score variability: 78.6% of registered nurses reported personally encountering patients harmed by quack nurses (QUACK26: *M* = 4.16, SD = 1.07), and 69.9% were aware of individuals in their region practising without qualifications (QUACK22: *M* = 3.84, SD = 1.28). The higher standard deviations for QUACK22 and QUACK26 reflect genuine variation in personal exposure to quackery, in contrast to the uniformly high endorsement of normative belief items.

**TABLE 3:** Quackery awareness and impact scale results (*N* = 618).

Item	*M* (SD)	Agreed (%)
QUACK24: Quackery damages public trust in nursing	4.82 (0.66)	97.1%
QUACK23: Quackery poses a serious threat to patient safety	4.77 (0.76)	96.1%
QUACK27: Strengthening NMCN enforcement will reduce quackery	4.71 (0.68)	96.6%
QUACK28: Public education can help communities identify quacks	4.69 (0.68)	96.9%
QUACK29: Professional nurses have a duty to expose quackery	4.68 (0.70)	96.1%
QUACK25: Regulatory checks are inadequate to prevent quackery	4.61 (0.81)	94.0%
QUACK26: I have personally encountered patients harmed by quack nurses	4.16 (1.07)	78.6%
QUACK22: Aware of unqualified individuals practising nursing in my region	3.84 (1.28)	69.9%
Overall scale	4.53 (0.58)	—

### 3.5 Inferential Analysis

#### 3.5.1 Chi‐Square Tests of Association

Chi‐square tests examined whether quackery awareness and encounters with patient harm differed by geopolitical region and facility type. Quackery awareness (QUACK22) and personal encounter with harm (QUACK26) were dichotomised as high (≥ 4) versus low (< 4). All expected cell counts exceeded 5, satisfying chi‐square assumptions.

As shown in Table 4, neither regional variation nor facility type was associated with quackery awareness or encounters with patient harm. Region × quackery awareness (*χ* ^2^(5) = 2.25, *p* = 0.814, Cramér's *V* = 0.060) and facility type × encounter with harm (*χ* ^2^(2) = 0.18, *p* = 0.915, Cramér's *V* = 0.017) both indicated no significant association with negligible effect sizes. Additional tests for region × overall quackery score high (*χ* ^2^(5) = 4.88, *p* = 0.431, *V* = 0.089) and facility × overall quackery score high (*χ* ^2^(2) = 0.28, *p* = 0.870, *V* = 0.021) were similarly non‐significant. Analytic Ns for facility‐type analyses (*N* = 612) are marginally lower than the full sample (*N* = 618) due to listwise exclusion of cases with missing facility type classification; region‐based analyses retained the full analytic sample of *N* = 618.

**TABLE 4:** Chi‐square tests of association.

Test	*N*	df	*χ* ^2^	*p*	Cramér's *V*
Region × quackery awareness (QUACK22 ≥ 4)	618^a^	5	2.25	0.814	0.060 (negligible)
Facility type × encounter with harm (QUACK26 ≥ 4)	612^b^	2	0.18	0.915	0.017 (negligible)
Region × overall quackery score (high)	618^a^	5	4.88	0.431	0.089 (negligible)
Facility type × overall quackery score (high)	612^b^	2	0.28	0.870	0.021 (negligible)

*Note:* Effect size interpretation: Cramér's *V* < 0.10 = negligible; 0.10–0.30 = small; 0.30–0.50 = moderate; > 0.50 = large (Cohen 1988).

^a^ *N* = 618 reflects the full analytic sample with complete data on region and the relevant quackery item.

^b^ *N* = 612 reflects the subsample with complete data on both facility type and the relevant quackery item, after listwise exclusion of 6 cases with missing facility type classification.

#### 3.5.2 Spearman's Rho Correlation Analysis

Spearman's rank correlations were computed between all subscale scores and key demographic variables. Table 5 presents the full correlation matrix. Ethical practice demonstrated the strongest positive association with quackery awareness (*ρ* = 0.421, *p* < 0.001, *n* = 616), followed by trust (*ρ* = 0.361, *p* < 0.001, *n* = 617). Trust and ethical practice were themselves strongly correlated (*ρ* = 0.578, *p* < 0.001). Professional values and professionalism scores were strongly intercorrelated (*ρ* = 0.526, *p* < 0.001), yet professionalism showed no significant correlation with quackery awareness (*ρ* = −0.033, *p* = 0.408). Years of nursing experience had a small but significant positive correlation with professionalism (*ρ* = 0.198, *p* < 0.001) but was unrelated to quackery awareness (*ρ* = −0.028, *p* = 0.489).

**TABLE 5:** Spearman's rho correlation matrix.

Variable	1	2	3	4	5	6
1. Professional values	—					
2. Professionalism	0.526***	—				
3. Trust	0.394***	0.278***	—			
4. Ethical practice	0.418***	−0.050	0.578***	—		
5. Quackery awareness	0.141***	−0.033	0.361***	0.421***	—	
6. Years of experience	0.069	0.198***	0.028	0.047	−0.028	—

*Note:* *n* varies 585–623.

*** *p* < 0.001.

#### 3.5.3 Multiple Linear Regression: Predictors of Quackery Awareness

Multiple linear regression examined predictors of the overall quackery awareness score (Table 6). The full model was statistically significant (*F*(11, 559) = 53.13, *p* < 0.001) and explained 51.1% of the variance (*R* ^2^ = 0.511, Adjusted *R* ^2^ = 0.501). Regression assumptions were verified prior to analysis: VIF values were all below 3.0, P–P plots indicated approximate normality of residuals, and residual scatterplots supported homoscedasticity. Residuals showed some non‐normality (skewness = −1.25, kurtosis = 7.83), consistent with a ceiling effect in the outcome variable; bootstrapped confidence intervals (1000 samples) were therefore computed and are reported alongside OLS estimates, confirming the robustness of findings.

**TABLE 6:** Multiple linear regression: Predictors of overall quackery awareness score (*N* = 571).

Predictor	*B*	SE	*β*	*p*	Bootstrapped 95% CI
Constant	1.065	0.209	—	< 0.001	[0.652, 1.478]
Ethical practice score	**0.541**	0.050	**0.541**	**< 0.001**	**[0.441, 0.641]**
Trust score	**0.215**	0.050	**0.215**	**< 0.001**	**[0.116, 0.314]**
Professionalism score	−0.004	0.031	−0.004	0.897	[−0.064, 0.056]
Years of experience	−0.001	0.002	−0.001	0.545	[−0.004, 0.002]
Region: SE vs. SW (ref.)	−0.003	0.051	−0.003	0.954	[−0.101, 0.096]
Region: NC vs. SW	−0.090	0.051	−0.090	0.079	[−0.191, 0.010]
Facility: Private vs. Public (ref.)	0.048	0.060	0.048	0.423	[−0.069, 0.165]
Facility: NGO vs. Public	−0.058	0.107	−0.058	0.587	[−0.266, 0.151]
Model: *R* ^2^ = 0.511; adjusted *R* ^2^ = 0.501; *F*(11, 559) = 53.13, *p* < 0.001					

*Note:* Reference categories: Region = South West (SW); Facility = Public. Bootstrapped 95% CIs based on 1000 samples. Bold values indicate statistically significant predictors (*p* < 0.001).

Abbreviations: NC, North Central; SE, South East.

Ethical practice was the strongest independent predictor (*β* = 0.541, *t* = 10.80, *p* < 0.001; bootstrapped 95% CI [0.441, 0.641]), followed by trust (*β* = 0.215, *t* = 4.34, *p* < 0.001; bootstrapped 95% CI [0.116, 0.314]). Neither professionalism score (*β* = −0.004, *p* = 0.897), years of experience (*β* = −0.001, *p* = 0.545), nor any regional dummy variable nor facility type were significant predictors.

## 4 Discussion

This study provides the first inferentially analysed examination of nursing quackery in Nigeria from the perspective of registered nurses, drawing on data from 618 participants recruited across all six geopolitical zones. The findings illuminate an important paradox: strong individual professionalism co‐exists with persistent system‐level failure.

### 4.1 High Professionalism in the Face of Systemic Failure

Perhaps the most striking finding is the consistently high scores across all professional domains—professional values (*M* = 4.76, SD = 0.44), professionalism (*M* = 4.76, SD = 0.58), trust (*M* = 4.53, SD = 0.59), and ethical practice (*M* = 4.84, SD = 0.59)—alongside near‐universal awareness that quackery threatens patient safety (96.1%) and damages public trust (97.1%). This confirms that nursing quackery in Nigeria is not a failure of individual professional commitment, but a failure of structural regulation. This aligns with Hall's (1968) Professionalism Theory, which situates professional standards as attributes of individuals embedded within an organisational and regulatory system. When systemic enforcement is weak, even the strongest individual professionalism cannot prevent the entry and persistence of unqualified practitioners (Amir‐Azodi et al. 2024; Aborode et al. 2021).

### 4.2 Quackery as a Uniform, Not Regional, Phenomenon

Quackery awareness did not vary significantly across Nigeria's geopolitical regions (*χ* ^2^(5) = 2.25, *p* = 0.814, Cramér's *V* = 0.060) or facility types (*χ* ^2^(2) = 0.18, *p* = 0.915, Cramér's *V* = 0.017). All effect sizes were negligible. This challenges a common assumption that quackery is primarily a rural or resource‐limited phenomenon. The regression analysis reinforces this conclusion: regional and facility‐type dummy variables did not independently predict quackery awareness after accounting for ethical practice and trust, compelling a shift from targeted, region‐specific interventions toward national‐level regulatory strategies.

### 4.3 Ethical Practice as Professional Vigilance

The strongest independent predictor of quackery awareness was ethical practice (*β* = 0.541, *p* < 0.001). Ethical practice, as measured here, captures registered nurses' commitment to reporting unqualified colleagues, adhering to NMCN guidelines, and refusing to participate in unethical practice. Its strong association with quackery awareness suggests that ethically oriented registered nurses are more likely to recognise, name, and report fraudulent practice—functioning as professional vigilance (Humphries et al. 2019; Rest 1986).

### 4.4 Trust and the Relational Dimension of Quackery Detection

Trust emerged as the second significant predictor (*β* = 0.215, *p* < 0.001) and was strongly correlated with ethical practice (*ρ* = 0.578, *p* < 0.001). The Trust Theory in Healthcare framework (Radwin and Cabral 2010) helps interpret this: registered nurses who hold a strong relational concept of trust are acutely sensitive to its violation. Quackery represents a fundamental betrayal of the trust placed in nursing by patients and society.

### 4.5 Non‐Significance of Professionalism and Experience

Professionalism scores and years of experience were not significant predictors of quackery awareness (*ρ* = −0.033 and −0.028, both *p* > 0.40). This likely reflects a ceiling effect: with professionalism scores clustered near the maximum (*M* = 4.76, range predominantly 4–5), insufficient variance exists to detect an association. This interpretation is supported by the non‐normal residuals in the regression model. Future research using broader‐range professionalism measures may reveal effects obscured here.

### 4.6 Implications for Nursing Practice and Policy

These findings have direct implications for nursing practice, education, and health policy in Nigeria.

#### 4.6.1 Implications for Nursing Practice

At the point of care, these findings call for heightened regulatory vigilance among registered nurses in identifying and challenging suspected nursing quackery within their own facilities and communities. Because ethical practice, not seniority or years of experience emerged as the strongest predictor of quackery awareness, nurse leaders and ward managers should actively cultivate ethical reflection and professional courage among nurses at every career stage, not only the most senior. Healthcare institutions should establish routine, ward‐level credential verification practices for all newly engaged nursing staff, supported by safe and protected reporting mechanisms that enable registered nurses to report suspected unqualified colleagues without fear of professional or social repercussion. Given that quackery awareness was statistically uniform across facility types and regions, these practices are equally relevant in public, private, and NGO‐run facilities, and across all six geopolitical zones, rather than being concentrated in perceived high‐risk settings.

#### 4.6.2 Implications for Nursing Education

Pre‐registration and continuing professional development curricula should embed ethics education that moves beyond rules‐based compliance toward the development of professional vigilance: the capacity to recognise, interpret, and act upon moral violations encountered in practice. Given the non‐significant relationship between years of experience and quackery awareness, such ethics education is equally relevant for newly qualified nurses as for senior practitioners, and should be integrated from the earliest stages of nurse training rather than treated solely as a continuing professional development topic. Nurse educators are also well placed to incorporate public health literacy content into community health teaching, equipping members of the public to verify nursing credentials and recognise the warning signs of fraudulent practice.

#### 4.6.3 Implications for Health Policy and Regulation

At the regulatory level, the NMCN requires substantially enhanced enforcement capacity: mandatory national licence verification systems accessible to both employers and the public, regular unannounced facility inspections, stronger penalties for employing unverified practitioners, and inter‐agency collaboration to investigate and prosecute fraudulent practice. Whistleblowing protection mechanisms should be formally established to operationalise the professional vigilance that ethically oriented registered nurses already demonstrate. Public awareness campaigns should help communities understand nursing qualifications, verify credentials through NMCN registers, and report suspected quackery, framed around the trust‐based nature of the nurse–patient relationship. Because the uniform national distribution of quackery rules out region‐specific intervention, these policy measures should be implemented as coordinated national strategies rather than localised pilot initiatives.

### 4.7 Strengths and Limitations

This study contributes a large cross‐sectional sample (*n* = 618) with geographic coverage spanning all six Nigerian geopolitical zones and diverse facility types, using validated instruments adapted to the Nigerian context. It is the first Nigerian study to apply inferential statistics including regression modelling to examine predictors of quackery awareness among registered nurses.

Several limitations must be acknowledged. First, the cross‐sectional design precludes causal inference. Second, the multistage sampling approach incorporated convenience sampling at Stage 3 within facilities, meaning the sample cannot be considered a statistically representative sample of all registered nurses in Nigeria; generalisability to all registered nurses, particularly those in hard‐to‐reach settings, should be treated with caution. Third, the online data collection modality may have introduced digital access bias, potentially underrepresenting older registered nurses and those in rural settings with limited internet connectivity; while smartphone penetration among Nigerian nursing professionals is increasing (Adeloye et al. 2022), selective participation remains a possibility. Fourth, technical duplicate prevention was not implemented; while instructional safeguards were in place, duplicate responses cannot be entirely excluded. Fifth, the sampling frame was constructed from professional networks rather than a complete register of practising nurses, which may overrepresent more professionally engaged registered nurses. Sixth, self‐reported data are vulnerable to social desirability bias, potentially inflating professional value and ethics scores and contributing to the ceiling effect observed in these subscales. Seventh, the regression model showed non‐normal residuals (skewness = −1.25, kurtosis = 7.83), consistent with a ceiling effect in the outcome variable; bootstrapped confidence intervals were therefore computed as a robustness check. Eighth, this study reflects the perspective of registered nurses only; future research should incorporate patient, administrator, and regulatory perspectives, which are being addressed in Phase 2 of this research programme.

Ninth, and importantly, this study reports registered nurses' ‘perceptions’ of nursing quackery rather than direct objective measurement of the phenomenon itself. Quackery awareness, exposure to harm, and professional values are all captured through self‐report rather than through direct observation, administrative records, or regulatory case data. Whilst self‐report is a well‐established and appropriate methodology for investigating professional perceptions and attitudes, the findings should be understood as reflecting how registered nurses experience and interpret quackery in their practice context, rather than as a direct epidemiological count of quackery incidents or perpetrators. Future research combining self‐report data with regulatory enforcement records, patient harm reports, and qualitative accounts would provide a more complete picture of the phenomenon.

## 5 Conclusion

This study demonstrates that Nigerian registered nurses exhibit strong professional values, deep ethical commitment, and high trust orientations, yet nursing quackery persists as a system‐level failure rooted in structural regulatory weaknesses, not a deficiency in professional standards. Ethical practice and trust were the primary drivers of quackery awareness, suggesting that ethically grounded registered nurses develop a form of professional vigilance that heightens their sensitivity to unsafe and fraudulent practice.

Addressing nursing quackery in Nigeria requires coordinated action—from the NMCN, healthcare institutions, educational bodies, and the public—to enforce existing regulations, promote ethical accountability, and protect the trust on which nursing care fundamentally depends. Failure to act is not merely a regulatory oversight; it is a betrayal of the patients whose safety registered nurses are entrusted to protect.

## Author Contributions

**Benjamin Olusola Ajibade:** conceptualisation, methodology, formal analysis, investigation, data curation, writing – original draft, writing – review and editing, project administration. **Isaiah Dada Owoeye:** methodology, validation, formal analysis, writing – review and editing, supervision. **Abiola Ogunniyi:** conceptualisation, resources, investigation, writing – review and editing, supervision. All authors read and approved the final manuscript.

## Funding

The authors havenothing to report.

## Disclosure

No generative artificial intelligence tools were used to generate study data, conduct statistical analysis, or produce the substantive scientific content, findings, or interpretations presented in this manuscript. The authors take full responsibility for the content, accuracy, and integrity of this work.

## Ethics Statement

Ethical approval was obtained from the Babcock University Health Research Ethics Committee, Nigeria (Ref—BUHREC 1127/24). All participants provided informed consent. Confidentiality and anonymity were maintained throughout; no identifying data were collected. Participants were informed of their right to withdraw at any stage without penalty. Data were stored securely in password‐protected systems accessible only to the research team.

## Conflicts of Interest

The authors declare no conflicts of interest.

## Supporting information


**File S1:** STROBE and CHERRIES reporting checklists. Completed Strengthening the Reporting of Observational Studies in Epidemiology (STROBE) checklist for cross‐sectional studies, and completed Checklist for Reporting Results of Internet E‐Surveys (CHERRIES). Both checklists map all required reporting items to their respective sections in the main manuscript. These checklists confirm that the study adheres to recommended standards for the reporting of cross‐sectional observational research and online survey methodology.

## Data Availability

The data that support the findings of this study are available from the corresponding author, Benjamin Ajibade (benjamin.ajibade@northumbria.ac.uk), upon reasonable request. Data are not publicly available due to participant confidentiality obligations and the absence of explicit participant consent for open data deposition at the time of data collection.
