# Supplementary material for: From Trust to Betrayal: Imposter Nurses and Nursing Quackery in Nigeria, Their Threat to Patient Safety, and the Role of Professionalism: A Cross‐Sectional Survey
Source: Nurs Open. 2026 Jul 17;13(7):e70707. doi: 10.1002/nop2.70707 (PMC13377210; doi:10.1002/nop2.70707)
Supplement: Supplementary file 1 — File S1: STROBE and CHERRIES reporting checklists. Completed Strengthening the Reporting of Observational Studies in Epidemiology (STROBE) checklist for cross‐sectional studies, and completed Checklist for Reporting Results of Internet E‐Surveys (CHERRIES). Both checklists map all required reporting items to their respective sections in the main manuscript. These checklists confirm that the study adheres to recommended standards for the reporting of cross‐sectional observational research and online survey methodology. [file NOP2-13-e70707-s001.docx]

**STROBE STATEMENT - CHECKLIST OF ITEMS FOR CROSS-SECTIONAL STUDIES**

Manuscript: Trust to Betrayal: Nursing Quackery in Nigeria — A Cross-Sectional Survey

Reference: von Elm E, Altman DG, Egger M, Pocock SJ, Gøtzsche PC, Vandenbroucke JP. The Strengthening the Reporting of Observational Studies in Epidemiology (STROBE) statement: guidelines for reporting observational studies. Lancet. 2007;370(9596):1453–1457.

| **Section/Topic** | **Item #** | **Recommendation** | **Reported on page/section #** |
| --- | --- | --- | --- |
| Title and abstract | 1 | (a) Indicate the study’s design with a commonly used term in the title or the abstract. (b) Provide an informative and balanced summary of what was done and what was found. | p.1, Title; p.2, Abstract |
| Introduction — Background/rationale | 2 | Explain the scientific background and rationale for the investigation being reported. | p.3, Introduction ¶¶1–6 |
| Introduction — Objectives | 3 | State specific objectives, including any prespecified hypotheses. | p.5, Aims and Objectives |
| Methods — Study design | 4 | Present key elements of study design early in the paper. | p.5, Study Design |
| Methods — Setting | 5 | Describe the setting, locations, and relevant dates, including periods of recruitment, exposure, follow-up, and data collection. | p.6, Study Setting; p.7-8, Data Collection |
| Methods — Participants | 6 | (a) Give the eligibility criteria, and the sources and methods of selection of participants. | p.6, Participants; Sample Size and Sampling |
| Methods — Variables | 7 | Clearly define all outcomes, exposures, predictors, potential confounders, and effect modifiers. Give diagnostic criteria, if applicable. | p.7, Instrument |
| Methods — Data sources/measurement | 8 | For each variable of interest, give sources of data and details of methods of assessment (measurement). Describe comparability of assessment methods if more than one group. | p.7, Instrument |
| Methods — Bias | 9 | Describe any efforts to address potential sources of bias. | p.7-8, Data Collection; p.8, Data Analysis |
| Methods — Study size | 10 | Explain how the study size was arrived at. | p.6, Sample Size and Sampling |
| Methods — Quantitative variables | 11 | Explain how quantitative variables were handled in the analyses. If applicable, describe which groupings were chosen and why. | p.8, Data Analysis |
| Methods — Statistical methods | 12 | (a) Describe all statistical methods, including those used to control for confounding. (b) Describe any methods used to examine subgroups and interactions. (c) Explain how missing data were addressed. (d) If applicable, describe analytical methods taking account of sampling strategy. (e) Describe any sensitivity analyses. | p.8, Data Analysis |
| Results — Participants | 13 | (a) Report numbers of individuals at each stage of study — eg numbers potentially eligible, examined for eligibility, confirmed eligible, included in the study, completing follow-up, and analysed. (b) Give reasons for non-participation at each stage. (c) Consider use of a flow diagram. | p.9, Participant Characteristics |
| Results — Descriptive data | 14 | (a) Give characteristics of study participants (eg demographic, clinical, social) and information on exposures and potential confounders. (b) Indicate number of participants with missing data for each variable of interest. | p.9–11, Results; Table 1 |
| Results — Outcome data | 15 | Report numbers of outcome events or summary measures. | p.11–14, Results; Tables 2–3 |
| Results — Main results | 16 | (a) Give unadjusted estimates and, if applicable, confounder-adjusted estimates and their precision (eg 95% confidence interval). Make clear which confounders were adjusted for and why they were included. (b) Report category boundaries when continuous variables were categorized. (c) If relevant, consider translating estimates of relative risk into absolute risk for a meaningful time period. | p.13–15, Inferential Analysis; Tables 4–6 |
| Results — Other analyses | 17 | Report other analyses done — eg analyses of subgroups and interactions, and sensitivity analyses. | p.15, Regression analysis with bootstrapped CIs |
| Discussion — Key results | 18 | Summarise key results with reference to study objectives. | p.16, Discussion (opening) |
| Discussion — Limitations | 19 | Discuss limitations of the study, taking into account sources of potential bias or imprecision. Discuss both direction and magnitude of any potential bias. | p.18–19, Strengths and Limitations |
| Discussion — Interpretation | 20 | Give a cautious overall interpretation of results considering objectives, limitations, multiplicity of analyses, results from similar studies, and other relevant evidence. | p.16–18, Discussion |
| Discussion — Generalisability | 21 | Discuss the generalisability (external validity) of the study results. | p.18-19, Strengths and Limitations |
| Other information — Funding | 22 | Give the source of funding and the role of the funders for the present study and, if applicable, for the original study on which the present article is based. | p.20, Declarations — Funding |

**Note:** An explanation and elaboration article discusses each checklist item and gives methodological background and published examples of transparent reporting. The STROBE checklist is best used in conjunction with this article (freely available on the websites of PLoS Medicine at http://www.plosmedicine.org/, Annals of Internal Medicine at http://www.annals.org/, and Epidemiology at http://www.epidem.com/). Information on the STROBE Initiative is available at http://www.strobe-statement.org.

**CHERRIES CHECKLIST — CHECKLIST FOR REPORTING RESULTS OF INTERNET E-SURVEYS**

Manuscript: Trust to Betrayal: Nursing Quackery in Nigeria — A Cross-Sectional Survey

Reference: Eysenbach G. Improving the quality of web surveys: the Checklist for Reporting Results of Internet E-Surveys (CHERRIES). J Med Internet Res. 2004;6(3):e34.

| **Item Category** | **Item #** | **Checklist Item** | **Reported on page/section #** |
| --- | --- | --- | --- |
| Design | 1 | Describe survey design (eg cross-sectional, longitudinal, panel). | p.5, Study Design — cross-sectional design clearly stated |
| IRB approval and informed consent | 2 | (a) Mention whether the study has been approved by an IRB. (b) Describe consent process. | p.9, Ethical Considerations |
| Data protection | 3 | If any personal information was collected or any...identifiable data, describe what mechanisms were used to protect data. | p.7-8, Data Collection — anonymisation and password protection described |
| Development and pre-testing | 4 | State how the survey was developed, including whether it was pre-tested. | p.7-8, Instrument — expert review and pilot study described |
| Recruitment process and description of the sample | 5 | Describe the process of recruitment, eg invitation by email or web page; survey method (eg internet, mail). | p.7-8, Data Collection — distribution channels described |
| Survey administration | 6 | (a) State the type of e-survey (eg distributed via mailing list or web/online survey). (b) Describe survey context (e.g. website look, advertisement). (c) Mention if the survey was incentivised. | p.7-8, Data Collection — Google Forms platform described |
| Response rates | 7 | (a) Calculate response rate (number completed/number invited). (b) Calculate completion rate. | p.6, Sample Size and Sampling — 98.6% response rate reported |
| Preventing multiple entries from the same individual | 8 | Describe steps taken to prevent multiple entries from the same individual. | p.7–8, Data Collection — instructional duplicate prevention described and limitation acknowledged |
| Analysis | 9 | (a) Describe handling of incomplete questionnaires. (b) Describe statistical correction techniques. | p.8, Data Analysis |
